# Ubiquitin-Related Proteostatic Programs in Cycling Fibroblast-Lineage Remodeling After Myocardial Ischemic Injury: A Hypothesis Informed by Single-Cell and Spatial Transcriptomics

**DOI:** 10.3390/ijms27146537

**Published:** 2026-07-22

**Authors:** Chengcheng Yi, Wenyuan Zheng, Jing Zhao, Weiting Cai, Junqian Wang, Li Song, Ming Bai, Zheng Zhang

**Affiliations:** 1 The First Clinical Medical College, Lanzhou University, Lanzhou 730000, China; dryicheng@hotmail.com (C.Y.);; 2 Heart Center, The First Hospital of Lanzhou University, Lanzhou 730000, China; 3 Key Laboratory for Cardiovascular Diseases of Gansu Province, The First Hospital of Lanzhou University, Lanzhou 730000, China

**Keywords:** myocardial ischemic injury, myocardial ischemia–reperfusion injury, ubiquitin–proteasome system, proteostasis, cardiac fibroblast, CCNB1, cell cycle, spatial transcriptomics, single-cell RNA sequencing, ventricular remodeling

## Abstract

Myocardial ischemia and reperfusion initiate spatially organized injury–repair programs that subsequently shape ventricular remodeling. Although cardiac fibroblasts are indispensable for scar formation, single-cell and spatial transcriptomic studies reveal temporally dynamic and regionally distinct fibroblast-lineage states. This critical narrative review integrates direct evidence from myocardial ischemia–reperfusion (I/R) with model-labeled evidence from permanent myocardial infarction, clinically heterogeneous human infarction, fibroblast-specific ubiquitin biology, cell-cycle regulation, and cardiac fibroblast atlases. A direct fibroblast I/R study identifies an HSP47–USP10–SMAD4 deubiquitination axis, whereas most other fibroblast ubiquitin–proteasome system (UPS) mechanisms derive from permanent infarction, non-ischemic cardiac stress, or in vitro systems. We propose that CCNB1-associated, G2/M-enriched cycling fibroblast-lineage states may impose heightened proteostatic demands within defined post-ischemic niches. The conceptual novelty is not that CCNB1 turnover or UPS activity is cardiac-specific; both are general features of proliferating cells. Rather, the framework asks whether fibroblast lineage, injury-model provenance, anatomical niche, temporal window, cell state, and substrate-specific UPS nodes jointly define proteostatic dependencies during post-ischemic remodeling. RNA-based ubiquitin-related signatures remain transcriptional proxies and do not directly quantify ubiquitinated substrates, ubiquitin-chain topology, enzyme activity, or proteasome flux. Resolving the proposed relationships will require spatial colocalization, protein-level and ubiquitin-remnant profiling, proteasome and ribosome assays, fibroblast-specific perturbation, and validation in human infarct tissue.

## 1. Introduction

Although timely reperfusion is indispensable after acute myocardial infarction, abrupt reoxygenation can trigger mitochondrial reactive oxygen species generation, calcium overload, and mitochondrial permeability transition [1,2,3]. Microvascular obstruction, endothelial dysfunction, and sterile inflammation can further extend tissue damage beyond the initial ischemic insult [4,5]. These acute events influence the subsequent transition from inflammation to repair, thereby shaping scar architecture and ventricular remodeling [6,7].

The post-ischemic myocardium constitutes a multicellular repair ecosystem in which cardiomyocytes, vascular cells, immune cells, and stromal cells exchange mechanical, metabolic, and inflammatory signals [8,9]. Fibroblast-lineage cells sense tissue injury, synthesize and remodel the extracellular matrix (ECM), communicate with neighboring cells, and stabilize the infarcted wall [10,11]. Although this response is essential for healing, sustained activation promotes excessive fibrosis, tissue stiffening, and adverse remodeling [12,13,14].

Single-cell transcriptomic studies have resolved activated, inflammatory, matrix-producing, and cycling fibroblast programs after myocardial infarction [15,16,17,18,19]. More broadly, single-cell RNA sequencing has become a central platform for defining cardiovascular cell states and disease-associated programs [20]. Spatial profiling has further localized stromal remodeling to infarct and border-zone niches in mouse and human hearts [21,22]. Together, these findings replace a binary quiescent-versus-activated model with a framework defined by cell state, time, and anatomical location.

Regulated protein turnover may provide a molecular bridge between fibroblast state and tissue remodeling. Ubiquitin ligases and deubiquitinating enzymes govern substrate stability and signaling [23,24,25], whereas the cardiac ubiquitin–proteasome system (UPS) maintains protein quality under basal and stress conditions [26,27,28]. Direct studies of myocardial ischemia–reperfusion injury (MIRI) have implicated defective ubiquitination–proteasome coupling, altered proteasome function, MARCH2, and USP25 in pathways associated with reperfusion injury [29,30,31,32]. Because mitotic progression requires the timely degradation of cyclin B1 and other short-lived regulators [33,34,35], a central unresolved question is whether cycling fibroblasts exhibit a distinctive dependence on UPS-mediated proteostasis.

### 1.1. Literature Search Strategy and Evidence Framework

This article was designed as a critical narrative mechanistic review rather than a systematic review or meta-analysis. PubMed was searched from database inception through 18 July 2026. The following search blocks were used: (1) (“myocardial ischemia-reperfusion” OR “ischemia-reperfusion” OR “myocardial infarction”) AND (“cardiac fibroblast” OR myofibroblast) AND (ubiquitin OR ubiquitination OR ubiquitylation OR deubiquitin* OR proteasome OR “E3 ligase”); (2) (“single-cell RNA sequencing” OR “single-cell transcriptom*” OR “spatial transcriptom*”) AND (“myocardial infarction” OR “ischemia-reperfusion”) AND (fibroblast OR myofibroblast); (3) (CCNB1 OR “cyclin B1” OR “G2/M”) AND (fibroblast OR “cardiac fibroblast”) AND (ubiquitin OR proteasome OR “APC/C”); and (4) (“ribosome biogenesis” OR “ribosomal RNA” OR rRNA OR “ribosome quality control”) AND (cardiac OR myocardial) AND (ischemia OR reperfusion OR fibroblast). Equivalent keyword combinations were adapted for Web of Science and Google Scholar, and reference lists and forward citations of key studies were checked.

Records were deduplicated by DOI and title, screened by title and abstract, and then assessed at full text for model provenance and relevance to the review questions. Studies were included in the evidence tables when they met at least one of the following criteria: (i) resolved cardiac fibroblast states after I/R or myocardial infarction by single-cell or spatial methods; (ii) demonstrated a mechanistic role for ubiquitination, a deubiquitinase (DUB), an E3 ubiquitin ligase, proteasomal degradation, or a ubiquitin-like modifier in cardiac fibroblasts; or (iii) established a foundational cell-cycle or proteostasis mechanism essential for interpreting the proposed model. Non-cardiac fibrosis studies, purely descriptive associations without defined fibroblast relevance, and studies with unclear injury-model provenance were excluded from the direct I/R category. Foundational reviews were retained for context but were not treated as primary mechanistic evidence. Because the review is narrative, the search was used for structured evidence mapping rather than PRISMA-style quantitative study accounting.

Evidence was classified into four non-equivalent categories and labeled consistently in the text and tables: (A) direct fibroblast evidence from controlled cardiac I/R; (B) fibroblast evidence from permanent infarction; (C) evidence from clinically heterogeneous human infarction; and (D) broader supporting evidence from non-ischemic cardiac models, whole-heart or cardiomyocyte studies, in vitro systems, or general cell biology. For example, APC/C-dependent degradation of cyclin B1 establishes biological plausibility [33,34,35] but does not demonstrate that ubiquitin-dependent CCNB1 regulation governs cardiac fibroblast remodeling. Likewise, a mechanism observed after permanent ligation was not interpreted as I/R-specific unless it was independently validated in a reperfusion model.

### 1.2. Article Type, Scope, Conceptual Novelty, and Knowledge Gaps

Existing reviews generally address myocardial I/R injury and cardiac fibrosis as separate topics [2,14], whereas others focus on the cardiac UPS, fibroblast heterogeneity, or ubiquitin-dependent mechanisms in cardiac fibrosis [26,27,36,37]. Methodological reviews have also examined single-cell and spatial transcriptomics [38,39]. The present article remains a Review and is positioned more specifically as a critical narrative mechanistic review with a hypothesis-generating integrative framework. It does not claim the exhaustive coverage or quantitative study accounting of a systematic review.

The conceptual novelty does not reside in proposing that cyclin B1 turnover or UPS activity is unique to the heart. CCNB1 is a conventional G2/M component, and ubiquitin-dependent turnover is required in proliferating cells generally [33,34,35]. Instead, the review asks whether applying six jointly necessary constraints can distinguish remodeling-relevant proteostatic dependencies from generic cell-cycle proteostasis. These constraints are cardiac fibroblast lineage, injury-model provenance, anatomical niche, temporal window, state identity, and substrate-specific UPS control. This context-constrained formulation connects independently established cardiac fibroblast UPS mechanisms with single-cell and spatial evidence without assuming convergence on CCNB1.

Accordingly, we first critically compare the temporal and spatial organization of fibroblast remodeling across controlled I/R, permanent infarction, and human infarction. We then evaluate direct and supporting fibroblast-specific ubiquitin mechanisms, cell-cycle control, and complementary ribosome-related demand before presenting the UPS–CCNB1 relationship as a testable, explicitly non-causal framework.

## 2. Myocardial Ischemic Injury as a Temporally Organized Repair Process

Ischemia suppresses oxidative phosphorylation, depletes ATP, and disrupts ion homeostasis [1,2]. Reperfusion can subsequently induce mitochondrial permeability transition, reactive oxygen species generation, and microvascular dysfunction [2,3,5]. Dying cardiomyocytes release danger-associated molecular patterns that activate innate immune signaling [6,40] and recruit neutrophils and monocyte-derived macrophages [8,41].

Effective repair depends on the resolution of excessive inflammation and coordinated activation of stromal programs. Lineage-tracing and single-cell studies show that fibroblasts proliferate, migrate, and acquire myofibroblast features after infarction [11,16,42]. These cells arise predominantly from resident cardiac lineages rather than bone marrow–derived populations [43]. Reparative fibroblasts express matrix-associated programs, including POSTN- and CTHRC1-related signatures [16,18]. ECM deposition stabilizes the injured wall [7,44], whereas persistent fibrotic activity promotes tissue stiffening, adverse remodeling, and heart failure [13,14,45].

The biological consequences of fibroblast proliferation therefore depend strongly on timing and context. Early expansion of reparative fibroblasts can facilitate scar formation [7,16,17], whereas persistent or spatially inappropriate cycling and matrix production may contribute to scar expansion and ventricular dysfunction [13,14,46]. Accordingly, therapeutic strategies should be defined by temporal window, cell state, and anatomical location rather than by indiscriminate suppression of fibroblast proliferation.

## 3. Fibroblast-State Heterogeneity: From Cell Types to Remodeling Programs

Cardiac fibroblasts are commonly annotated by Pdgfra, Tcf21, Dcn, Lum, Col1a1, and Col1a2, whereas activated myofibroblast-like states often express Postn, Fn1, Acta2, and Cthrc1 [10,18,36]. Although useful for annotation, these markers do not define immutable cell types. Integrated atlases and high-resolution cardiac transcriptomic studies instead reveal dynamic, partially overlapping cellular programs [19,47].

Collectively, single-cell studies support four conclusions. First, cardiac injury remodels stromal, vascular, and immune compartments in concert [15,17]. Second, infarcted hearts contain inflammatory and matrix-producing fibroblast states [16,18] alongside conserved proliferative programs [19,48]. Third, cycling states express genes such as Mki67, Top2a, Cdk1, Ube2c, Birc5, and Ccnb1 [15,19,49]; however, these cell-cycle modules should not be assumed to identify differentiated myofibroblasts unless activation markers are demonstrated in the same cells. Fourth, spatial context distinguishes programs in the infarct border zone from phenotypically similar states in remote myocardium [21,22].

We therefore organize fibroblasts by remodeling program rather than by a fixed subtype catalog. Inflammatory programs coordinate with immune cells, matrix-producing programs build and organize the scar, and cycling programs expand portions of the stromal compartment. Cycling and myofibroblast programs may overlap, but they should remain analytically distinct until co-expression, lineage, and functional evidence demonstrate convergence. Selective protein turnover is a biologically plausible regulatory layer because cell-cycle entry, mitosis, and exit require the timed degradation of short-lived proteins. Representative state-resolved studies are summarized in Table 1.

Together, these studies define cycling fibroblasts through transient cell-cycle modules or proliferation-marker expression, whereas spatial studies localize activated and matrix-producing programs to infarct and border-zone niches. Most studies in Table 1 used permanent infarction or clinically heterogeneous myocardial infarction rather than controlled ischemia–reperfusion. None directly quantified fibroblast-lineage ubiquitinated substrates, ubiquitin-chain topology, E3 ligase or deubiquitinase activity, or proteasome flux. Thus, the evidence supports a state-resolved transcriptomic framework but not a fibroblast-specific biochemical UPS mechanism in MIRI.

## 4. Ubiquitination and Proteostasis in Post-Ischemic Cardiac Remodeling

Ubiquitination is a reversible post-translational modification in which E1 enzymes activate ubiquitin, E2 enzymes catalyze its transfer, and E3 ligases confer substrate specificity; deubiquitinating enzymes (DUBs) remove or remodel ubiquitin chains [23,24,25,51]. Depending on chain architecture and cellular context, ubiquitin can direct proteasomal degradation or regulate trafficking, autophagy, inflammatory signaling, and cell-cycle progression [23,52,53].

The cardiac UPS preserves protein quality under basal and stress conditions [26,27,28]. I/R increases the burden of oxidized, misfolded, and damaged proteins [26,54]. Experimentally enhancing proteasome function can attenuate I/R injury, whereas disrupted coupling between ubiquitination and proteasomal degradation exacerbates myocardial damage [29,30]. MARCH2 and USP25 have been linked to NLRP3-related I/R pathways in whole-heart or cardiomyocyte contexts [31,32]. Importantly, direct fibroblast evidence is provided by transient LAD occlusion/reperfusion and hypoxia/reoxygenation experiments in which HSP47 recruited USP10, promoted SMAD4 deubiquitination, and aggravated fibroblast activation and fibrosis [55].

This HSP47–USP10–SMAD4 study establishes that a substrate-specific DUB mechanism can operate in fibroblasts after controlled I/R [55], but it does not link that mechanism to a CCNB1-associated cycling state. Other cardiac fibroblast UPS mechanisms summarized below arise chiefly from permanent infarction, non-ischemic stress, or in vitro systems. They inform substrate and pathway selection but are not treated as evidence of an I/R-specific CCNB1 mechanism.

## 5. Cardiac Fibroblast-Specific Ubiquitin Mechanisms and Candidate Regulators

The UPS comprises several regulatory tiers: E3 ligases confer substrate selectivity, DUBs reverse or remodel ubiquitin signals, and the proteasome removes many polyubiquitinated proteins [23,24,25,56]. Cardiac UPS function, cardiac E3 ligases, and the expanding role of ubiquitin pathways in cardiac fibrosis have been reviewed previously [37,57,58]. Here, primary studies are reclassified by injury model and by the directness of fibroblast evidence so that mechanistic support is not conflated with I/R specificity.

Only the HSP47–USP10–SMAD4 axis currently provides direct fibroblast evidence in a controlled cardiac I/R model within the studies identified by this search [55]. Permanent MI studies support WWP2–SMAD2, OTUD4–PFKFB3, FBXL8–Snail1, ASPP1–OTUB1–p53, HSPA12A–USP10–p53, USP7–KLF7–GATA3, UCHL1–GRP78, SPOP–RACK1, and UCK2/UCKL1–TRIM21–Smurf2 mechanisms [59,60,61,62,63,64,65,66,67]. Angiotensin II-stimulated fibroblasts support USP2–β-catenin–cyclin D1 and UCHL1–NF-κB regulation [68,69], whereas hiPSC-derived cardiac fibroblasts support a BAG3–HSP70–CHIP–TGFBR2 degradation axis [70]. FAT10-mediated proteasomal SMAD3 degradation involves a ubiquitin-like modifier rather than canonical ubiquitination [71]. An earlier UCHL1 study in PDGF-BB-stimulated cardiac fibroblasts involved suppressed autophagic, rather than proteasomal, p21 degradation and was therefore retained as boundary evidence [72]. These studies establish fibroblast-relevant substrate mechanisms but remain heterogeneous in species, injury model, lineage specificity, and endpoint.

None of these primary studies directly demonstrates ubiquitin-dependent regulation of CCNB1 in a spatially resolved cardiac fibroblast state. Their value is twofold. They identify experimentally supported fibroblast UPS nodes and substrates, and they define competing profibrotic or protective mechanisms. These mechanisms should be tested for overlap with the CCNB1-associated cycling program rather than presumed to belong to it. Candidate prioritization should integrate model-matched spatial localization, protein-level evidence, substrate engagement, and fibroblast-specific perturbation.

### Evidence from Cardiac Fibroblasts and Model-Specific Boundaries

Table 2 summarizes primary evidence for ubiquitin-dependent or ubiquitin-like mechanisms in cardiac fibroblasts. The table distinguishes controlled I/R, permanent infarction, non-ischemic stimulation, and in vitro systems. E3 ligases and DUBs remain attractive because they confer substrate selectivity and reversibility; nevertheless, mRNA abundance alone cannot establish enzyme activity, substrate engagement, ubiquitin-chain topology, or proteasome flux.

## 6. Interpretive Boundaries of Transcriptomic Evidence

Transcriptomic enrichment should not be equated with biochemical activity. RNA sequencing does not measure ubiquitinated substrates, ubiquitin-chain topology, E3 ligase or DUB activity, or proteasome flux. Accordingly, a cluster enriched for UPS-related transcripts should not be described as exhibiting increased ubiquitination without supporting protein-level or biochemical evidence. Quantitative proteomics and ubiquitin-remnant profiling provide suitable approaches for such validation [73,74,75].

Throughout this review, the term “ubiquitin-related transcriptional program” denotes the coordinated expression of genes involved in ubiquitin conjugation, deubiquitination, proteasomal degradation, or ubiquitin-dependent signaling. This deliberately constrained terminology can nominate pathways for experimental testing but cannot identify the modified substrate, chain type, enzyme activity, or degradation rate.

The same interpretive constraint applies to CCNB1. Cyclin B1 is a conventional G2/M regulator whose mitotic destruction depends on APC/C [33,34,35,76,77]; this chemistry is neither cardiac- nor fibroblast-specific. The proposed model becomes remodeling-specific only when a CCNB1-associated cycling program is demonstrated within cardiac fibroblast-lineage cells, in a defined injury model, anatomical niche, and temporal window, together with direct evidence for the relevant UPS enzyme, substrate, and biological consequence. In cardiac transcriptomic data, a CCNB1-associated fibroblast state should therefore be interpreted as a candidate cycling program, not as evidence of cyclin B1 protein positivity, CCNB1-driven fibrosis, or direct ubiquitin-dependent regulation.

### rRNA and Ribosome Biogenesis as a Complementary Biosynthetic-Demand Axis

Ribosomal RNA forms the structural and catalytic core of the ribosome, and ribosome biogenesis is a major determinant of translational capacity [78]. Proliferating and matrix-producing fibroblasts may therefore experience heightened biosynthetic demand, although transcriptomic data alone cannot establish increased rRNA synthesis. In cardiac I/R, γ2-AMPK-dependent suppression of pre-rRNA transcription and ribosome biogenesis reduced endoplasmic reticulum stress and injury [79]. In primary cardiac fibroblasts, the RNA polymerase I inhibitor CX-5461 suppressed activation, proliferation, and myofibroblast differentiation primarily through p53 activation rather than reduced ribosome biogenesis [80]. The cardiac consequences of nucleolar stress, however, remain context-dependent [81].

Ribosome biology also intersects directly with ubiquitin-dependent proteostasis. Poly(A)-capture RNA sequencing does not directly quantify mature rRNA [82], and many droplet-based single-cell workflows likewise focus on polyadenylated transcripts. When ribosomes stall or collide, the E3 ligase ZNF598 recognizes collided ribosomes and ubiquitinates ribosomal proteins, thereby initiating ribosome-associated quality-control signaling [83]. Future studies should therefore quantify 47S pre-rRNA, 18S/28S rRNA, nucleolar activity, nascent translation, ribosome collisions, and quality-control proteins in spatially defined fibroblast populations.

## 7. Proteostatic Requirements of Cycling Fibroblast-Lineage States

Cycling fibroblast-lineage cells divide while simultaneously responding to cytokines, mechanical strain, matrix stiffness, oxidative stress, and a substantial ECM biosynthetic burden. These convergent demands necessitate tightly regulated proteostasis.

Four mechanisms support this premise. First, APC/C-dependent turnover coordinates cyclins and mitotic regulators [33,34,76,77], while SCF and other ubiquitin–proteasome mechanisms provide additional cell-cycle control [84,85,86]. Second, matrix production increases the demand for protein folding and quality control [12,36]. Third, fibroblast activation entails substantial cytoskeletal remodeling [87]. Fourth, NF-κB and inflammasome signaling are regulated by ubiquitination and deubiquitination [23,25,31,32]. These mechanisms establish biological plausibility but do not demonstrate that a single cycling fibroblast state simultaneously expresses a differentiated myofibroblast program.

Accordingly, the proposed relationship is not a simple linear effect of ubiquitination on CCNB1 and is not presented as a cardiac-specific cell-cycle mechanism. The testable proposition is narrower: within a defined post-ischemic fibroblast lineage, region, and time window, substrate-specific UPS nodes may alter the entry, maintenance, or resolution of a CCNB1–CDK1-associated state and thereby influence scar architecture. Direct and supporting fibroblast studies identify several candidate UPS axes and boundary mechanisms (Table 2), but none has yet been mapped to a CCNB1-associated state. This gap distinguishes the novel integrative question from established general cell-cycle proteostasis.

### Interpretive Value and Limitations of CCNB1

CCNB1 is most informative when interpreted within a broader G2/M module that includes MKI67, TOP2A, CDK1, UBE2C, BIRC5, AURKA, and AURKB. Co-expression of these genes identifies cycling fibroblast programs in single-cell atlases [15,19]. CCNB1 alone neither defines a stable cell type nor establishes a causal mechanism. Cyclin B1 abundance changes rapidly through coordinated synthesis, localization, and APC/C-dependent degradation [33,34,35], and increased CCNB1 mRNA may simply reflect the cell-cycle phase. We therefore use the term CCNB1-associated cycling fibroblast-lineage state for the transcriptomic program and reserve CCNB1 protein-positive for cells validated by protein-level or spatial protein assays.

## 8. Spatial Organization of Cycling Fibroblast-Lineage Remodeling

Anatomical location fundamentally shapes the biological significance of a fibroblast state. Human spatial multi-omics has delineated distinct infarct, border, remote, and vascular-associated myocardial niches [21], while mouse spatial studies have identified the emergence of a transcriptionally distinct border zone after infarction [22]. Spatial transcriptomics thus restores anatomical context to cell states inferred from dissociated single-cell data [38,39].

Spatial profiling can determine whether CCNB1-associated fibroblast signatures concentrate in the border zone or other regions of active matrix remodeling. It can also assess their proximity to macrophage-rich, vascular, or collagen-dense niches and determine whether UPS-related transcription overlaps with the cycling program. Human infarct maps provide disease-specific spatial evidence [21], whereas healthy adult heart atlases provide a reference for cell-state annotation [88].

**Figure 1:**
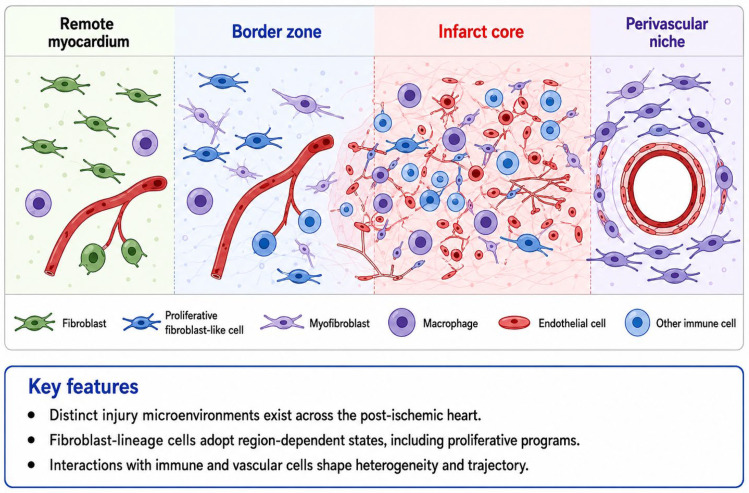
Spatial organization of fibroblast remodeling niches after myocardial ischemic injury. The infarct core, border zone, remote myocardium, and perivascular regions provide distinct inflammatory, mechanical, vascular, and extracellular matrix cues that shape fibroblast states. The schematic summarizes spatial context rather than a fibroblast-intrinsic mechanism involving the ubiquitin–proteasome system [15,21,22].

**Figure 2:**
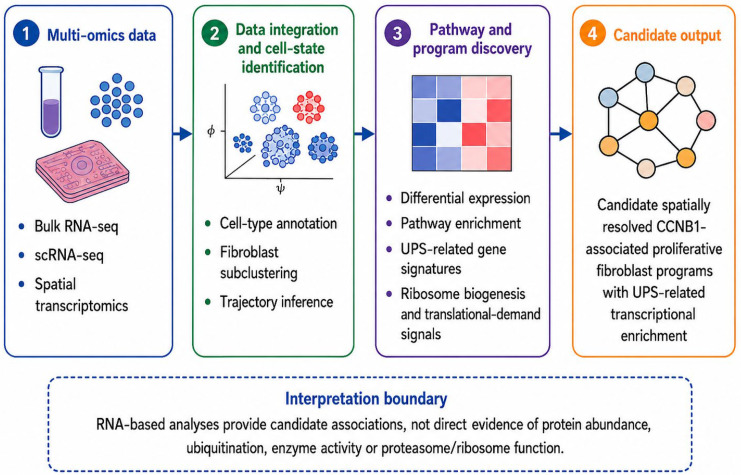
Proposed analytical workflow from datasets to candidate programs. Colors distinguish analytical stages and schematic elements and do not encode quantitative values. This schematic illustrates how bulk RNA sequencing, single-cell RNA sequencing, fibroblast subclustering, and spatial transcriptomics could be integrated to nominate a spatially resolved CCNB1-associated cycling fibroblast program with UPS-related transcriptional enrichment. It is a conceptual workflow; no new multi-omics analysis was performed in this review. The proposed steps prioritize biochemical and functional testing but do not establish regulatory direction or causality.

Spatial data nevertheless have important limitations. Spot-based platforms may combine neighboring cell types, and deconvolution depends on the selected reference; resolution also varies across spot-based, sequencing-based, and imaging-based platforms [38,39]. Apparent colocalization should therefore be validated by multiplex immunofluorescence, RNA in situ hybridization, spatial proteomics, or microdissection followed by proteomic and ubiquitin-specific assays. Figure 1 summarizes the regional remodeling landscape, whereas Figure 2 presents a proposed analytical workflow showing how integrated transcriptomic analyses could nominate candidate programs at the cell-state and pathway levels.

## 9. Intercellular and Extracellular Regulation of Fibroblast States

Fibroblast states are shaped by signals from neighboring cells and the ECM. Cardiomyocytes and immune cells provide stress, cytokine, and growth-factor cues [6,8,9]. Macrophage populations change dynamically during cardiac repair [41], and spatial studies reveal evolving cardioimmune niches during lesion resolution [50]. Matrix architecture influences fibroblast fate [89], while TGF-β/Smad signaling exerts fibroblast-specific effects that may oppose those in other cardiac cell types within the infarcted heart [90,91,92]. Collectively, these inputs coordinate fibroblast proliferation, matrix production, and local inflammation.

Single-cell ligand–receptor analyses can nominate TGF-β, IL-6-family, chemokine, collagen–integrin, fibronectin, and osteopontin signaling axes [21,93]. Such inference is valuable for prioritization but does not demonstrate signaling flux. Within this framework, the local injury niche serves as an upstream source of both cell-cycle entry and proteostatic demand.

### Integrated Mechanistic Hypothesis

**Figure 3:**
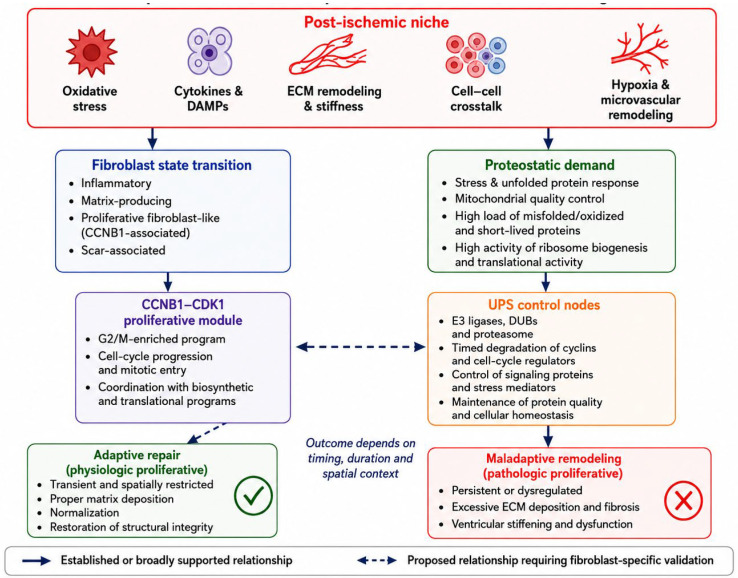
Integrated model of UPS-related proteostasis and the CCNB1–CDK1 cycling module in post-ischemic fibroblast remodeling. Colors distinguish cell types, tissue components, and molecular modules and do not encode quantitative values. Solid arrows denote established or broadly supported relationships, whereas dashed arrows indicate proposed fibroblast-specific links. The direct fibroblast I/R mechanism is supported by [55], and additional model-specific UPS mechanisms are summarized in Table 2. The remodeling consequences of the cycling program may depend on timing, duration, and spatial context [7,16,29,33,34,35].

As summarized in Figure 3, direct evidence from controlled I/R supports fibroblast HSP47–USP10–SMAD4 signaling [55]. Additional cardiac fibroblast UPS mechanisms derive from permanent MI models [59,60,61,62,63,64,65,66,67], non-ischemic models [68,69], and in vitro systems [70]. FAT10-mediated proteasomal degradation is classified as evidence involving a ubiquitin-like modifier [71], whereas UCHL1-associated autophagic p21 degradation is retained as boundary evidence at the UPS–autophagy interface [72]. Separately, single-cell and spatial studies resolve cycling and regionally organized fibroblast programs [15,16,17,18,19,20,21,22,50]. The integrated hypothesis is that selected UPS nodes may support, constrain, or terminate a CCNB1–CDK1-associated cycling module in fibroblast states defined by injury model, region, and time. No current study demonstrates convergence of these two evidence streams; therefore, Figure 3 links them with proposed rather than established relationships.

## 10. Translational Implications: Precision over Broad Inhibition

The near-term value of this framework lies in mechanistic discovery and tissue stratification. If validated in human myocardium, a CCNB1-associated fibroblast signature could identify regions of active stromal expansion. Transcriptomic association alone, however, does not justify considering either CCNB1 or the UPS an immediate therapeutic target.

Therapeutic timing is critical. Early fibroblast proliferation can support scar stabilization [7,16], whereas persistent cycling or fibrotic activation may contribute to scar expansion and ventricular dysfunction [13,14]. Any intervention would therefore need to be cell-state-specific, spatially targeted, and confined to an appropriate temporal window.

A practical next step is to relate spatial fibroblast states to infarct age, collagen architecture, immune composition, ventricular remodeling, and clinical outcomes. Candidate ubiquitin regulators should then be evaluated through ubiquitinomics and fibroblast-specific perturbation rather than inferred from transcript abundance alone.

## 11. Validation Roadmap from Association to Mechanism

**Figure 4:**
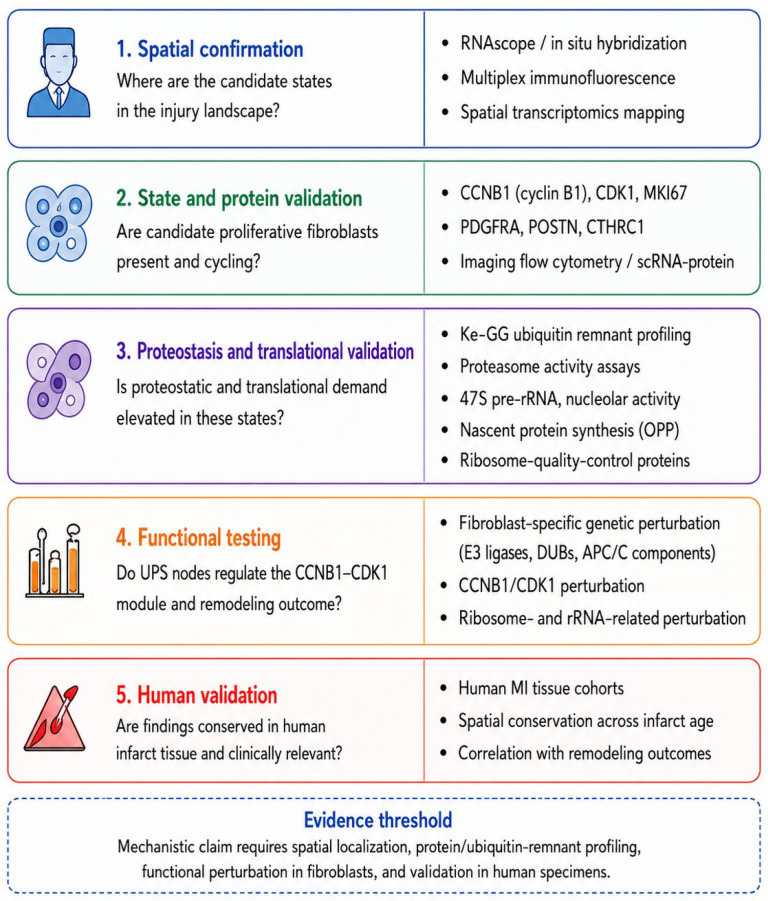
Validation roadmap from association to mechanism. Candidate states should first be confirmed spatially and at the protein level, followed by ubiquitin-remnant profiling, proteasome assays, rRNA and translation assays, and fibroblast-specific perturbation. Human myocardial infarction tissue is required to assess conservation and clinical relevance [21,73,74,75,88].

The validation sequence in Figure 4 directly addresses the relationships proposed in Figure 3. First, multiplex immunofluorescence or RNA in situ hybridization should determine whether CCNB1 or other proliferation markers colocalize with PDGFRA, POSTN, COL1A1, ACTA2, or CTHRC1 in defined injury regions. Protein-level measurements should then establish whether the RNA-defined state corresponds to a genuinely proliferative fibroblast population.

Subsequent studies should use ubiquitin-remnant profiling, quantitative proteomics, and proteasome assays to identify altered substrates and pathways, ideally in sorted or spatially enriched fibroblasts [73,74,75]. Ribosome-related measurements should include pre-rRNA abundance, nucleolar activity, nascent translation, and ribosome quality-control readouts [79,80,82,83]. Candidate E3 ligases, DUBs, APC/C components, proteasome regulators, ribosome quality-control factors, and cell-cycle mediators should then be perturbed specifically in fibroblasts. Finally, human infarct specimens should be used to assess conservation, spatial localization, and clinical relevance [21,88].

## 12. Evidence Matrix

The outstanding questions and evidence domains supporting this hypothesis are summarized in Box 1 and Table 3.

Box 1 **Outstanding questions.**

- Are CCNB1-associated cycling fibroblast-lineage states conserved between mouse and human myocardial infarction?
- At what reperfusion time points do these states emerge, peak, and resolve?
- Which E3 ligases, DUBs, and proteasome regulators are active in cycling fibroblast-lineage cells?
- Do ubiquitin-related programs support adaptive scar formation, maladaptive fibrosis, or both, depending on timing and spatial context?
- Can spatially restricted niches of cycling fibroblasts predict ventricular remodeling or progression to heart failure?

## 13. Limitations

Several limitations constrain interpretation. First, this is a critical narrative review; the structured search improves reproducibility but does not provide the exhaustive coverage or quantitative study accounting of a systematic review. Second, permanent coronary ligation, transient I/R, and clinical myocardial infarction differ in injury kinetics, residual perfusion, inflammatory exposure, and scar evolution. We therefore distinguish direct fibroblast I/R evidence, permanent MI evidence, heterogeneous human evidence, and broader mechanistic support throughout the text and tables. Third, transcriptomic signatures do not measure protein modification, enzyme activity, proteasome flux, rRNA synthesis, or translation. Fourth, cycling signatures may reflect a transient cell-cycle phase rather than a stable fibroblast or myofibroblast identity. Fifth, spatial platforms differ in resolution and may combine neighboring cell types [38,39].

Within the literature identified here, the HSP47–USP10–SMAD4 axis is the clearest fibroblast-specific mechanism directly established in a controlled cardiac I/R model [55]. Additional UPS-relevant mechanisms in cardiac fibroblasts have been reported in permanent MI models [59,60,61,62,63,64,65,66,67] and non-ischemic models [68,69]. Further mechanistic support derives from cardiac fibroblast studies conducted in vitro [70]. FAT10-mediated proteasomal degradation [71] is classified as evidence involving a ubiquitin-like modifier, whereas UCHL1-associated autophagic p21 degradation [72] is retained as boundary evidence at the UPS–autophagy interface (Table 2). Most I/R evidence on proteasome function and ribosome biogenesis derives from cardiomyocytes or whole-heart analyses. No study directly links a defined UPS substrate mechanism to a spatially resolved CCNB1-associated fibroblast state. Differences in species, ischemia duration, reperfusion interval, lineage definition, comorbidity, treatment, and tissue availability further limit translation to human disease [21,88,94].

## 14. Conclusions

This critical narrative review supports three evidence-tiered conclusions. First, single-cell and spatial studies establish that fibroblast-lineage cells enter inflammatory, matrix-producing, and cycling programs, but most such atlases derive from permanent infarction or clinically heterogeneous human infarction. Second, a direct controlled I/R study establishes fibroblast HSP47–USP10–SMAD4 signaling [55], whereas the remaining cardiac fibroblast evidence derives from permanent MI models, non-ischemic models, and in vitro systems. FAT10-mediated degradation is treated as evidence involving a ubiquitin-like modifier, whereas autophagic p21 degradation is retained as boundary evidence at the UPS–autophagy interface (Table 2). Third, no study yet demonstrates that these mechanisms regulate a CCNB1-associated fibroblast state after reperfusion.

The central conclusion is therefore intentionally circumscribed. CCNB1 turnover and UPS activity are general features of proliferating cells, not cardiac-specific novelties. The proposed contribution is a context-constrained framework that asks whether fibroblast lineage, injury-model provenance, anatomical niche, temporal window, state identity, and substrate-specific UPS control jointly determine proteostatic dependencies during post-ischemic remodeling. Testing this framework requires model-matched spatial colocalization, protein and rRNA validation, ubiquitin-specific proteomics, lineage or fate mapping, fibroblast-specific perturbation, and confirmation in human tissue.

## Figures and Tables

**Table 1:** Representative single-cell and spatial transcriptomic studies defining fibroblast states after myocardial infarction or ischemic injury. Model type and reperfusion status are specified because permanent infarction and ischemia–reperfusion are not interchangeable. Abbreviations: F-Cyc, cycling fibroblast cluster; I/R, ischemia–reperfusion; LAD, left anterior descending coronary artery; LV, left ventricle; MI, myocardial infarction; RNA-FISH, RNA fluorescence in situ hybridization; sc/snRNA-seq, single-cell/single-nucleus RNA sequencing; UPS, ubiquitin–proteasome system.

Study	Species, Model, and Reperfusion Status	Sampling Time	Fibroblast Populations/States	Cycling Markers/Program	Spatial Localization	Direct UPS Evidence
Farbehi et al. [15]	Mouse; permanent LAD ligation MI; Pdgfra-GFP reporter; no reperfusion	Days 3 and 7	Activated, inflammatory, cycling and myofibroblast states	F-Cyc expressed Ccnb2, Cdk1, and Mki67; cycling was identified transcriptomically	WIF1-positive F-WntX cells localized to the day-3 border zone; direct spatial localization of F-Cyc was not established	No direct UPS evidence; state mapping was transcriptomic and histologic
Forte et al. [17]	Mouse; permanent coronary ligation MI in C57BL/6J and 129S1/SvImJ strains; no reperfusion	Baseline and days 1, 3, 5, 7, 14, and 28	Early injury-response, activated myofibroblast and late scar-associated stromal states	Transient cycling/mitotic program; no discrete CCNB1-defined population was emphasized	Infarct-derived interstitial cells; no spatial transcriptomic localization	No fibroblast-specific UPS assay
Ruiz-Villalba et al. [18]	Mouse; permanent LAD ligation MI; Col1a1-GFP reporter; no reperfusion	Days 7, 14 and 30	CTHRC1-positive reparative fibroblasts	Cthrc1, Postn, and collagen programs; proliferation was not the primary classifier	Predominantly associated with the infarct scar	No direct UPS measurement
Kuppe et al. [21]	Human clinical MI and control hearts; multiple LV zones; reperfusion status was heterogeneous or unresolved	Distinct post-MI pathological stages	Fibroblast-to-myofibroblast trajectories and POSTN-positive myofibroblast states	No discrete cycling or CCNB1-defined fibroblast population was specifically reported	Infarct, border, remote, and vascular-associated niches; myofibroblasts localized near SPP1-positive macrophages	No direct UPS evidence; RNA- and chromatin-based state mapping only
Calcagno et al. [22]	Mouse; permanent LAD ligation; sc/snRNA-seq, spatial transcriptomics, and RNA-FISH; no reperfusion	Early hours to days after injury	Matricellular fibroblasts localized near border-zone cardiomyocytes	Activation/ECM programs; no CCNB1-defined cycling state was reported	Enriched near the infarct edge and emerging border zone	No direct UPS evidence; state and regional mapping only
Chan et al. [50]	Adult mouse; two injury models: transient LAD ligation followed by reperfusion and LV cryoablation; scRNA-seq integrated with high-resolution targeted spatial transcriptomics	Days 1, 3, 7, 28, and 56 (scRNA-seq); days 7 and 28 (high-resolution spatial profiling)	Quiescent and Ccl2-positive fibroblasts; cycling, IFN-positive, Hp-positive, Dkk2-positive, and Postn-positive/Thbs4-positive myofibroblasts; mature Il1rapl1-positive matrifibrocytes	Day-3-enriched cycling myofibroblasts with high G2/M/cell-cycle gene expression; expression declined from day 7 onward, with macrophage–fibroblast interactions suppressing cell-cycle activity during scar maturation	High-resolution spatial maps (Cryo hearts) localized fibroblast states across infarct/fibrotic, border, and remote zones; the LAD I/R model was profiled by scRNA-seq, with spatial relevance supported by model comparison and published Visium data	No direct UPS measurement; the study resolves spatiotemporal cell-cycle dynamics and macrophage–fibroblast control of proliferation
Patrick et al. [19]	Integrated mouse and human cardiac fibrosis datasets, including mouse MI; not a dedicated I/R comparison	Model-dependent; includes early and later MI	Conserved activated, matrix-producing, proliferative, and matrifibrocyte programs	MKI67/TOP2A/CDK1/UBE2C/CCNB1-associated cycling module	Cross-study integration; spatial annotation was not uniform across datasets	No; transcript-level evidence only
Nie et al. [48]	Mouse; reanalysis of one public MI and one sham scRNA-seq sample (GSM4040774 and GSM4040775); reperfusion status was not specified in the reanalysis	Single public MI–sham comparison; sampling time was not reported in the reanalysis	Postn-positive fibroblast population with a high fibrosis score	C1 Postn-positive cells were largely in G1; proliferative potential was inferred and supported by in vitro Postn knockdown rather than by a discrete G2/M cluster	Not directly spatially resolved	No direct UPS measurement

**Table 2:** Direct and supporting ubiquitin-dependent mechanisms and boundary evidence relevant to cardiac fibroblast remodeling. Model provenance is shown explicitly to prevent extrapolation from permanent infarction or non-ischemic systems to controlled ischemia–reperfusion. Abbreviations: Ang II, angiotensin II; CF, cardiac fibroblast; DUB, deubiquitinating enzyme; H/R, hypoxia/reoxygenation; hiPSC, human induced pluripotent stem cell; I/R, ischemia–reperfusion; LAD, left anterior descending coronary artery; MI, myocardial infarction; UPS, ubiquitin–proteasome system.

Study/Axis	Model and Provenance	Fibroblast-Specific Evidence	Mechanistic Finding	Relevance and Boundary
Xie et al. [55]; HSP47–USP10–SMAD4	Mouse transient LAD occlusion/reperfusion; H/R CFs; direct I/R	Fibroblast-selective HSP47 manipulation and fibroblast USP10 loss	HSP47 recruited USP10 and promoted SMAD4 deubiquitination, fibroblast activation, and fibrosis	Direct fibroblast I/R UPS mechanism; no evidence for a CCNB1-associated state or spatial colocalization
Chen et al. [59]; WWP2–SMAD2	Mouse permanent MI and pressure overload; primary CFs	Fibroblast-enriched WWP2 isoform and CF perturbation	WWP2 modulated SMAD2 signaling and pathological fibrosis	Direct CF mechanism, but not reperfusion-specific and not linked to CCNB1
Wang et al. [60]; OTUD4–PFKFB3	Mouse permanent LAD ligation; TGF-β-stimulated neonatal rat CFs	CF knockdown/overexpression and substrate-stability assays	OTUD4 deubiquitinated and stabilized PFKFB3, promoting glycolysis and fibrosis	Permanent MI metabolic mechanism; cannot be assigned to controlled I/R
Li et al. [61]; FBXL8–Snail1	Mouse permanent MI; cultured rat CFs	CF substrate and ubiquitination assays	FBXL8 targeted Snail1 for ubiquitin–proteasome degradation and limited fibrosis	Permanent MI E3 ligase mechanism; no I/R evidence or evidence for a CCNB1-associated state
Li et al. [62]; ASPP1–OTUB1–p53	Mouse permanent MI with Postn-lineage conditional deletion; myofibroblasts	Myofibroblast-targeted genetics and mechanistic CF assays	ASPP1 interfered with OTUB1-dependent p53 stabilization, facilitating p53 degradation and fibrosis	Strong lineage-targeted evidence from permanent MI; Postn targeting does not cover all fibroblast states
Mao et al. [63]; HSPA12A–USP10–p53	Mouse permanent MI; primary CFs	CF-specific expression and scaffold/interaction experiments	HSPA12A scaffolded USP10–p53 signaling and limited glycolysis, activation, and fibrosis	Protective mechanism in permanent MI; shares USP10 but a different substrate from direct I/R study [55]
Yang et al. [64]; USP7–KLF7–GATA3	Mouse permanent MI with fibroblast/myofibroblast-specific USP7 deletion; primary CFs	Cell-specific genetics and substrate deubiquitination assays	USP7 stabilized KLF7, promoted GATA3 transcription, and drove myofibroblast activation	Strong permanent MI fibroblast mechanism; not controlled I/R and not linked to CCNB1
Lei et al. [65]; UCHL1–GRP78	Mouse permanent MI; TGF-β-stimulated CFs	Post-MI pharmacological inhibition plus CF knockdown and interaction assays	UCHL1 promoted GRP78 ubiquitination and degradation and supported fibrosis	Permanent MI cardiac fibroblast mechanism; systemic inhibitor limits lineage specificity
Yang et al. [66]; SPOP–RACK1	Mouse permanent LAD ligation; TGF-β-stimulated CFs	CF gain/loss experiments and substrate-specific ubiquitination assays	SPOP promoted RACK1 ubiquitination and degradation, activating SMAD3 signaling	Permanent MI E3 adaptor mechanism; no controlled I/R validation
Zhou et al. [67]; UCK2/UCKL1–TRIM21–Smurf2	Mouse permanent MI; TGF-β-stimulated human CFs; human ischemic tissue	Fibroblast/myofibroblast knockdown and molecular scaffold assays	UCK2/UCKL1 recruited TRIM21 to ubiquitinate and degrade Smurf2, sustaining SMAD3 signaling	Recent permanent MI mechanism with human support; reperfusion was not tested
Xu et al. [68]; USP2–β-catenin–cyclin D1	Ang II-stimulated CFs in vitro; non-ischemic	Primary CF gain/loss experiments	USP2 stabilized β-catenin and increased cyclin D1 and activation	Supports DUB–proliferation coupling; no infarction or I/R validation
Gong et al. [69]; UCHL1–NF-κB	Ang II-infused mouse and primary CFs; non-ischemic	CF proliferation and pathway perturbation with systemic UCHL1 inhibition	UCHL1 and NF-κB formed a profibrotic feed-forward relationship	Cardiac fibroblast support without infarction, reperfusion, or a defined ubiquitinated substrate
Morsink et al. [70]; BAG3–HSP70–CHIP–TGFBR2	hiPSC-derived CFs in vitro; no ischemic injury model	Human CF-like cells with molecular perturbation	BAG3–HSP70–CHIP controlled TGFBR2 ubiquitination and degradation	Support from a human cell system; lacks infarction, reperfusion, and spatial validation in tissue
Chen et al. [71]; FAT10–SMAD3	Mouse permanent myocardial ischemia; primary and hiPSC-derived CFs	CF functional assays with systemic FAT10 manipulation	The ubiquitin-like modifier FAT10 promoted proteasomal SMAD3 degradation and reduced fibrosis	Evidence involving a ubiquitin-like modifier, not canonical ubiquitination; no reperfusion
Zhang et al. [72]; UCHL1–p21	Mouse transverse aortic constriction; PDGF-BB-stimulated neonatal rat CFs; non-ischemic	Cardiac expression and primary CF proliferation/autophagy assays	UCHL1 suppressed autophagic p21 degradation, increased p21, and restrained proliferation	Boundary evidence at the UPS–autophagy interface; no UPS-mediated p21 degradation or I/R validation

**Table 3:** Evidence matrix supporting a spatially informed hypothesis linking ubiquitin-related transcriptional programs to cycling fibroblast-lineage remodeling after myocardial ischemic injury.

Evidence Domain	Representative Evidence	Relevance to This Review	Directness to a Fibroblast-Specific Ischemic-Injury Mechanism	Key Knowledge Gap
MIRI and repair biology	Reperfusion injury integrates mitochondrial damage, inflammation and ECM remodeling [2,6,7].	Defines the signals that generate remodeling niches.	Strong but not fibroblast-specific.	How injury timing selects cycling fibroblast-lineage states.
Fibroblast heterogeneity	Single-cell and lineage studies identify activated, matrix-producing and cycling fibroblast programs [15,16,18,19].	Supports state-specific rather than uniform fibroblast remodeling.	Strong for fibroblast biology; mechanistic interpretation remains model-dependent.	Durability and function of cycling states after I/R.
Spatial transcriptomics	Human and mouse studies map infarct and border-zone stromal niches [21,22].	Provides anatomical context for candidate states.	Moderate; dependent on platform resolution.	Single-cell spatial protein validation is required.
UPS biology	Ubiquitin-chain signaling, E3-ligase specificity, and DUB activity are well established [23,24,25].	Provides molecular plausibility for proteostasis–cell-cycle coupling.	Strong general biology; indirect for fibroblast MIRI.	Define substrates and enzyme activity within fibroblast states.
UPS in myocardial I/R injury	Proteasome coupling, MARCH2, and USP25 are linked to I/R pathways [29,30,31,32]; HSP47–USP10–SMAD4 is a direct fibroblast I/R mechanism [55].	Supports UPS relevance to reperfusion injury and identifies one fibroblast-specific substrate axis.	Direct for fibroblast I/R only for [55]; the remaining evidence is whole-heart or cardiomyocyte based.	Determine whether the HSP47–USP10–SMAD4 axis or other UPS nodes intersect with spatially defined cycling states.
CCNB1 and cell-cycle turnover	Cyclin B1 destruction and G2/M progression depend on APC/C and related ubiquitin machinery [33,34,35,84].	Explains why a CCNB1/CDK1 module requires timed protein turnover.	Mechanistic, but indirect.	Test this machinery in cardiac fibroblast-lineage cells.
Validation technologies	Quantitative proteomics and ubiquitin-remnant profiling can test RNA-derived hypotheses [73,74,75].	Defines the transition from association to mechanism.	Required for causal inference.	Integrate with lineage tracing and human tissue.
rRNA, ribosome biogenesis and ribosome quality control	Cardiac I/R studies link suppressed ribosome biogenesis to reduced injury [79]; in fibroblasts, RNA polymerase I inhibition exerts p53-dependent antifibrotic effects [80], while ZNF598 mediates collision-associated quality control [83].	Connects translational load, p53-dependent stress signaling, and UPS-mediated ribosome quality control.	Evidence is cardiomyocyte-based, general, or non-I/R; [80] does not establish reduced rRNA synthesis as the antifibrotic mechanism, and fibroblast-specific I/R evidence remains limited.	Measure pre-rRNA, translation and ribosome quality control in spatially defined fibroblasts.
Integrated UPS–cell-cycle model	Fibroblast UPS substrate mechanisms and boundary evidence (Table 2) and state-resolved cycling programs [15,16,17,18,19,20,21,22,50] are independently supported.	Defines a testable intersection conditioned on lineage, injury model, region, time, state, and substrate.	Hypothesis-generating; no study has demonstrated convergence on CCNB1 in cardiac fibroblasts.	Test overlap, directionality, and remodeling consequences using model-matched fibroblast-specific perturbation.

## Data Availability

No new data were created or analyzed in this study. Data sharing is not applicable to this article.
